# ATP Hydrolysis by α‐Synuclein Amyloids is Mediated by Enclosing β‐Strand

**DOI:** 10.1002/advs.202508441

**Published:** 2025-10-16

**Authors:** Lukas Frey, Fiamma Ayelen Buratti, Istvan Horvath, Shraddha Parate, Ranjeet Kumar, Roland Riek, Pernilla Wittung‐Stafshede

**Affiliations:** ^1^ ETH Zürich Institute of Molecular Physical Science Zürich 8092 Switzerland; ^2^ Department of Life Sciences Chalmers University of Technology Gothenburg 412 96 Sweden; ^3^ Department of Chemistry Rice University Houston TX 77005 USA

**Keywords:** alpha‐synuclein, amyloid, ATP, catalytic activity, cryo‐EM

## Abstract

Pathological amyloids, like those formed by α‐synuclein in Parkinson's disease, are recently found to catalyze the hydrolysis of model substrates in vitro. Here it is reported that the universal energy molecule ATP is another substrate for α‐synuclein amyloid chemical catalysis. To reveal the underlying mechanism, the high‐resolution cryo‐EM structure of the amyloids in the presence of ATP is solved. The structure reveals a type 1A amyloid fold with an additional β‐strand involving residues 16‐22 that wraps around the ATP, creating an enclosed cavity at the interface of the protofilaments. Mutations of putative ATP‐interacting residues in the cavity and the additional β‐strand showed that replacing any one of Lys21, Lys23, Lys43, Lys45, and Lys60 with Ala reduced amyloid‐mediated ATPase activity. High‐resolution structural analysis of Lys21Ala α‐synuclein amyloids in the presence of ATP reveals the same fold as wild‐type α‐synuclein amyloids but without the extra β‐strand and with ATP oriented differently. It is concluded that positively‐charged side chains, along with ordering of the N‐terminal part into a β‐strand, enclosing the cavity, are essential parameters governing ATP hydrolysis by α‐synuclein amyloids. Amyloid‐catalyzed ATP hydrolysis may hamper ATP‐dependent rescue systems near amyloid deposits in vivo.

## Graphical abstract

Pathological amyloids have been considered chemically inert until recently. Here it is shown that α‐synuclein amyloids catalyze the hydrolysis of the universal energy molecule, ATP. The cryo‐EM structure of the complex reveals an additional β‐strand (purple) that encloses the ATP‐binding site. Within the cavity, several lysine residues are required for chemical reactivity.

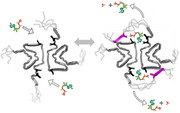

## 1 Introduction

Amyloids are polymers of monomeric protein units non‐covalently assembled through β‐strands arranged perpendicularly to the fibril long axis, forming a cross‐β structure.^[^ ^1^ ^]^ The cross‐β arrangement is the basis of all amyloid fibers, but the exact packing (fold, topology) of the β‐strand arrangement in each perpendicular plane varies widely among amyloid systems; even the same polypeptide can adopt different amyloid polymorphs depending on conditions and other unknown factors.^[^ ^2^ ^]^ Although several functional amyloids are known (e.g., bacterial curli),^[^ ^3^ ^]^ amyloid formation is mostly connected to human neurodegenerative diseases, such as Parkinson's disease (PD) and Alzheimer's disease, and type 2 diabetes.^[^ ^4^ ^]^ Here, proteins with normal functions as monomers start (for some unknown reason) to assemble into amyloids, resulting in both loss of monomer function as well as gain of toxicity coupled to the assembly processes. The resulting amyloid fibrils are often considered end products with intermediate species (so‐called oligomers) formed during aggregation, as most toxic to cells. Deleterious gain‐of‐function coupled to amyloid formation are mitochondrial dysfunction, oxidative stress, metabolic changes, metal ion dyshomeostasis, genome instability, and eventually cell death.^[^ ^5^ ^]^

PD is the second most common neurodegenerative disorder and the most frequent movement disorder today, for which there is only symptomatic treatment.^[^ ^6^ ^]^ Amyloid fibers of the protein α‐synuclein (αSyn) constitute the major content of pathological intraneuronal inclusions, Lewy bodies, found in dopaminergic neurons in PD patient brains.^[^ ^7^ ^]^ In accord, duplications, triplications, and point mutations in the αSyn gene, enhancing expression and aggregation, are linked to familial PD cases.^[^ ^8^ ^]^ Although soluble αSyn oligomers are proposed to be most toxic,^[^ ^9^ ^]^ it is clear that αSyn amyloid fibrils themselves are toxic too and can be transmitted from cell to cell and cross the blood‐brain barrier.^[^ ^10^ ^]^

Amyloid toxicity has been attributed to the ability to seed new amyloids, to translocate between cells, to deteriorate membranes, to be a sink for functionally relevant proteins by binding, and to sterically block cellular functions. Amyloids were considered chemically inert until we showed that αSyn amyloids catalyzed hydrolysis of ester and phosphoester bonds in vitro.^[^ ^11^ ^]^ In addition, we detected distinct chemical alterations of important metabolites in neuronal cell lysates (devoid of proteins; only small molecules present) upon incubation with αSyn amyloids.^[^ ^12^ ^]^ This enzyme‐like behavior of αSyn amyloids, which has been paralleled by similar results on amyloid‐β (linked to Alzheimer's disease) and glucagon (hormone, unknown link to disease) amyloids,^[^ ^13^ ^]^ implies that many amyloid systems may have yet‐unknown chemical reactivities^[^ ^11a^ ^]^ as also explored in the context of new materials and the origin of life.^[^ ^14^ ^]^

Lewy pathology, i.e., amyloids, is also found in the nuclei of cells, and our earlier work showed αSyn monomers to interact with DNA.^[^ ^15^ ^]^ When we extended this to amyloids, we found that αSyn amyloid interactions with DNA promote strand breaks in the DNA.^[^ ^16^ ^]^ Thus, the chemical reactivity of αSyn amyloids may contribute to the noted widespread DNA damage observed in PD patients. As the damage was proposed to involve cleavage of phosphoester bonds in the DNA backbone, and we had observed dephosphorylation of model phosphoesters, it appeared possible that αSyn amyloids would also hydrolyze other phosphoester bond molecules, such as adenosine‐5'‐triphosphate (ATP). ATP is the primary energy currency in biological systems, and its hydrolysis drives most metabolic and biosynthetic reactions in cells.

Neurons have disproportionately high energy demands compared to other organs but lack energy fuel storage (such as fatty acids and glucogen). In contrast to many other cells, neurons must continuously produce ATP from glucose to meet the cellular demands and maintain energy homeostasis.^[^ ^17^ ^]^ Most ATP in neuronal cells is made in mitochondria^[^ ^18^ ^]^ and then transported to other cell compartments. The total ATP concentration in brain cells can be in the millimolar range^[^ ^19^ ^]^ while the free ATP concentration is many orders of magnitude lower. Under conditions of stress, such as DNA damage and dysfunctional mitochondria, it may decrease drastically.^[^ ^18^, ^20^ ^]^ Decline in brain ATP levels has been connected to both Alzheimer's and PD.^[^ ^21^ ^]^ There is evidence that αSyn amyloids perturb mitochondria, resulting in lower ATP production,^[^ ^22^ ^]^ and ATP itself has been found to bind to αSyn monomers and modulate its aggregation kinetics.^[^ ^23^ ^]^ Many questions around temporal and spatial variations of cellular ATP levels remain.^[^ ^24^ ^]^

Here, we combine biochemical, biophysical, computational, and structural methods to probe the interaction between αSyn amyloids and ATP. We report that αSyn amyloids display catalytic activity toward ATP hydrolysis in vitro. Upon solving a high‐resolution structure of the ATP‐amyloid complex by cryo‐EM, we identify the ATP binding site along with an adapted type 1A αSyn amyloid fold that includes an additional β‐strand in the N‐terminus. Point‐mutations of selected αSyn residues confirm the proposed ATP binding site. The high‐resolution structure of a mutated αSyn amyloid, lacking ATPase activity, reveals a similar type‐1A fold but without an additional β‐strand and the ATP bound in a different orientation. In silico analysis supports the observed distinct ATP positions in the two αSyn amyloid structures. We propose that ATP depletion by αSyn amyloid hydrolysis may disturb the local energy balance in neuronal cells. From a structural perspective, our results demonstrate that small‐molecule binding can induce additional ordering and thus alter the exposed surface of already formed amyloids.

## 2 Results

### 2.1 αSyn Amyloids Hydrolyze ATP

**Figure 1:**
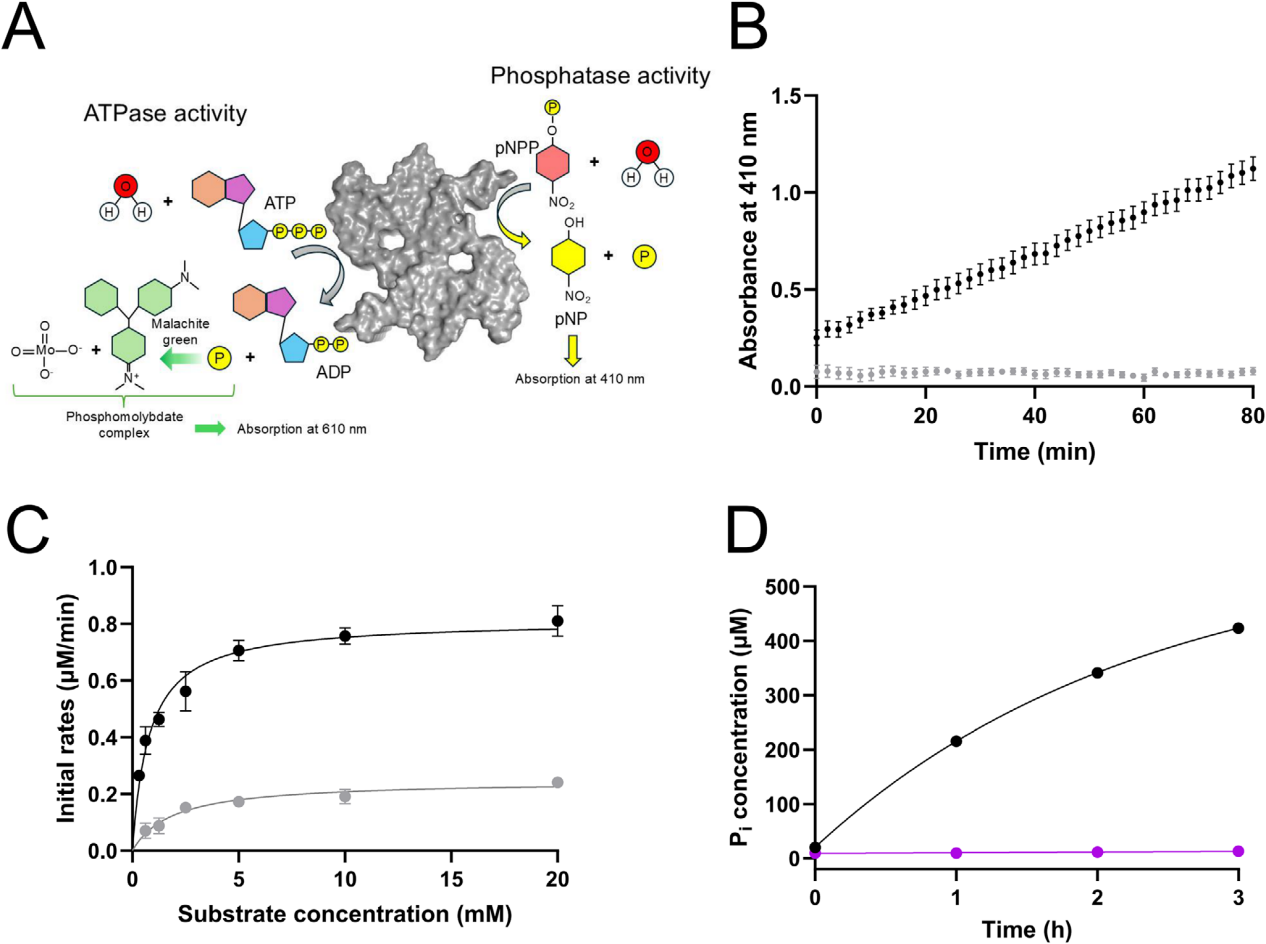
ATP binds the same site as pNPP and is hydrolyzed by WT aSyn amyloids. A) panel showing chemical reactions for pNPP hydrolysis and ATP malachite green assay. B) Phosphatase activity of 40 µm WT αSyn amyloids in the absence (black) or presence of 5 mm ATP (gray). C) Michaelis–Menten plot of initial reaction rates of 20 µm WT αSyn amyloids as a function of pNPP concentrations in the absence (black) and presence of 2.5 mm ATP (gray), with Michaelis–Menten fit as solid curve. Error bars, mean ± SD (N = 3). D) ATPase activity of 40 µm WT αSyn amyloids (black) or WT αSyn monomers (purple) in the presence of 1 mm ATP.

Since WT αSyn amyloids were found to hydrolyze the model phosphoester compound para‐nitrophenyl orthophosphate (pNPP),^[^ ^11^ ^]^ we decided to assess if this amyloid chemical activity could be extended to the biological substrate ATP, which also contains phosphoester bonds. First, we probed the putative inhibitory effect of ATP on the pNPP reaction that is conveniently detected as an increase in 410 nm absorbance over time (**Figure** **1**A). Whereas αSyn amyloids display pNPP hydrolysis without ATP, the presence of 5 mm ATP completely blocks pNPP hydrolysis by the amyloids (Figure 1B). At a lower ATP concentration, 2.5 mm, there was some pNPP hydrolysis detected, and Michaelis–Menten parameters could be derived. In the presence of 2.5 mm ATP, the K_M_ value increased (from 0.8 to 1.9 mm) and the k_cat_ value decreased (from 0.02 to 0.006 min^−1^) as compared to in the absence of ATP (Figure 1C). This observation suggests that ATP binds to αSyn amyloids and competes with pNPP for the same sites. However, this experiment does not show whether ATP is hydrolyzed or not by the αSyn amyloids.

To directly address ATP hydrolysis by αSyn amyloids, we used the Malachite Green assay to detect the generation of free phosphate (Figure 1A). A significant increase in phosphate concentration with time was observed upon mixing 1 mm ATP with WT αSyn amyloids, but not when ATP was incubated with αSyn monomers (Figure 1D). Thus, ATP binding to αSyn amyloids triggers its hydrolysis. After 3h of incubation, we detect 400 µm free phosphate in the presence of amyloids, with kinetics displaying an initial phosphate generation rate of ≈195 µm h^−1^ at these conditions. This value corresponds to a k_cat_ of 0.08 min^−1^ (assuming 1 mm ATP is a condition giving maximal rate), which is in the same ballpark as the detected pNPP hydrolysis rate constant (0.02 min^−1^, see above). As 10 times more free phosphate is generated within 3 h than there are αSyn monomers in the amyloids, the reaction is catalytic (notably, at 3 h, the reaction is still ongoing).

### 2.2 Novel Features in the Structure of the ATP‐Amyloid Complex

**Figure 2:**
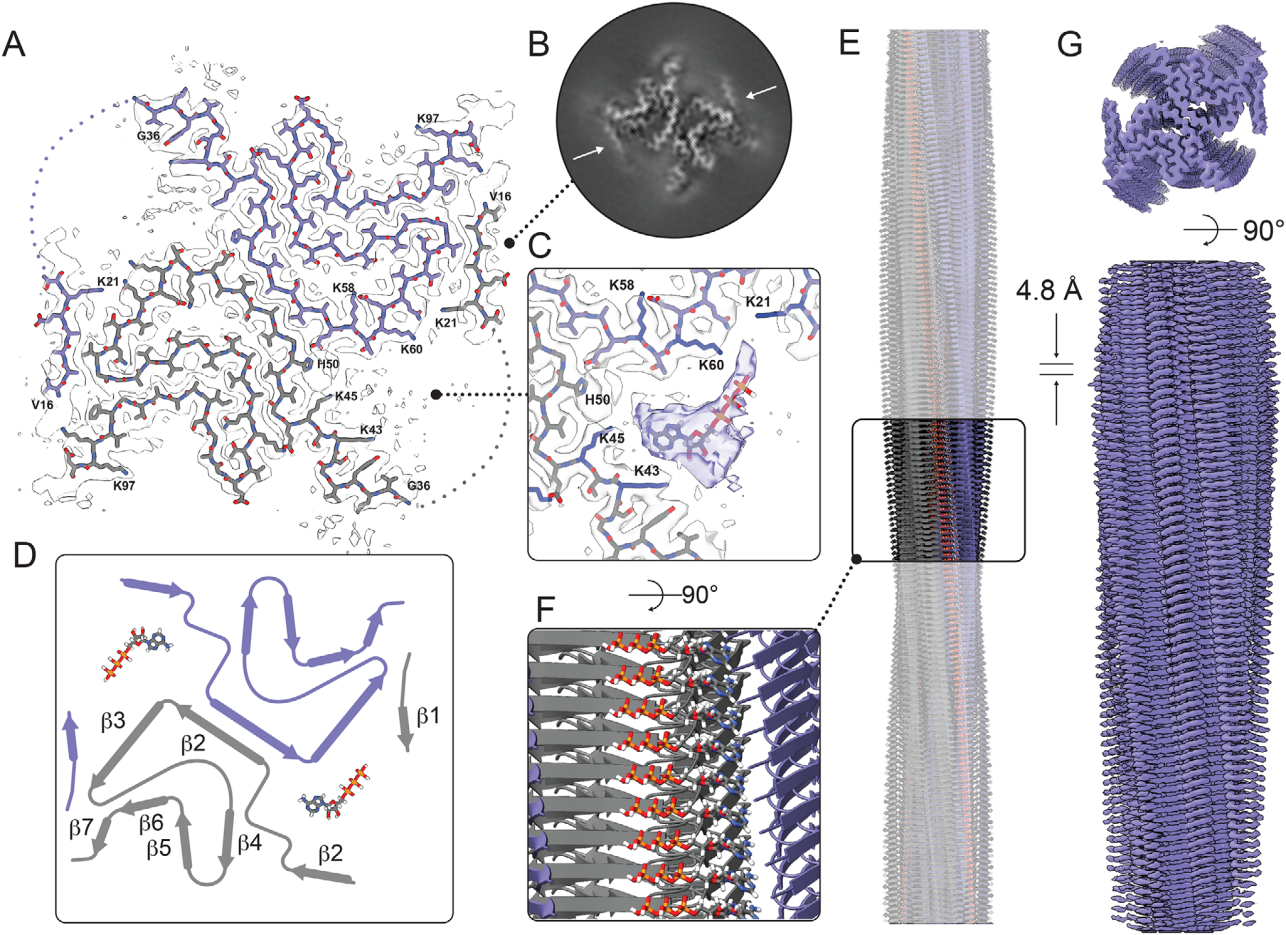
Cryo‐EM structure of WT αSyn amyloids in the presence of ATP. A) Postprocessed cryo‐EM density map overlayed with the atomic model, where the dotted line indicates the missing residues to connect the newly formed β‐strand by residues V16 to K21 (white arrows). B) Cryo‐EM density map from the 3D refinement; the white arrows indicate the additional β‐strand V16 to K21. C) Chimeric cryo‐EM density map combining the post‐processed map (grey) with the 3D refinement map (purple). ATP was modeled into the density using ChimeraX. The nucleotide moiety of ATP is found deepest inside the pocket, with the phosphate tail directed toward the V16–K21 region. D) Schematic representation of the type 1A αSyn polymorph bound to ATP, illustrating the overall polymorphism of the amyloid fibril. E) (F, zoom in) Representative illustration of ATP position on the surface of the amyloid fibril. G) Post‐processed cryo‐EM density map of the ATP‐bound fibril, used to build the atomic model.

To investigate the interaction between WT αSyn and ATP at a molecular level, we turned to cryo‐EM and solved the structure of WT αSyn amyloids in the presence of ATP to a resolution of 3.08 Å (**Figure** **2**; Figure S1 and Table S1, Supporting Information). In parallel, we confirmed that WT αSyn amyloids in the absence of ATP displayed a type 1A polymorph fold, similar to previously reported structures 6h6b and 6a6b (Figure S2 and Table S1, Supporting Information, final resolution 3.20 Å). The high‐resolution structure of WT αSyn amyloids in the presence of 1 mm ATP also shows a type 1A‐fold, as expected since the ATP was added after fibril formation. Interestingly, however, there is an extra density corresponding to the N‐terminus forming a β‐strand involving residues 16‐21 (Figure 2A–D). Furthermore, there is an additional density for ATP in the cavity between the two protofilaments (Figure 2C). The ATP density is not fully resolved, and thus its exact orientation cannot be stated with certainty. The diffuse density rather indicates that ATP binds in several similar binding poses dynamically. The cavity in which ATP is found contains several lysine residues: Lys43, Lys45, Lys60, as well as Lys21 and Lys23 (predicting its position based on the partially resolved residue 22) in the additionally ordered β‐strand. The sole histidine in the polypeptide, His50, is also deep inside this cavity (Figure 2A–C). The five positively charged lysine residue side chains orchestrate thereby the binding of the highly negatively charged ATP molecule.

The structural difference between WT αSyn amyloids with and without bound ATP was also evident from amyloid seeding ability toward fresh αSyn monomers (ATP‐bound amyloids were more efficient), and proteinase K degradation (ATP‐bound amyloids showed increased resistance) (Figure S3, Supporting Information).

### 2.3 Lysine Residues are Important for Amyloid Chemical Reactivity

In earlier work, using pNPP, we found that His50Ala mutated αSyn amyloids did not show any pNPP hydrolysis.^[^ ^11^ ^]^ However, that mutation may result in αSyn amyloids with a different polymorph, so it is hard to determine the underlying reason for the loss of activity. To address the putative ATP site with more conservative mutations, we turned to the lysines in the ATP cavity, whose positive charges may form electrostatic interactions with the ATP.

**Figure 3:**
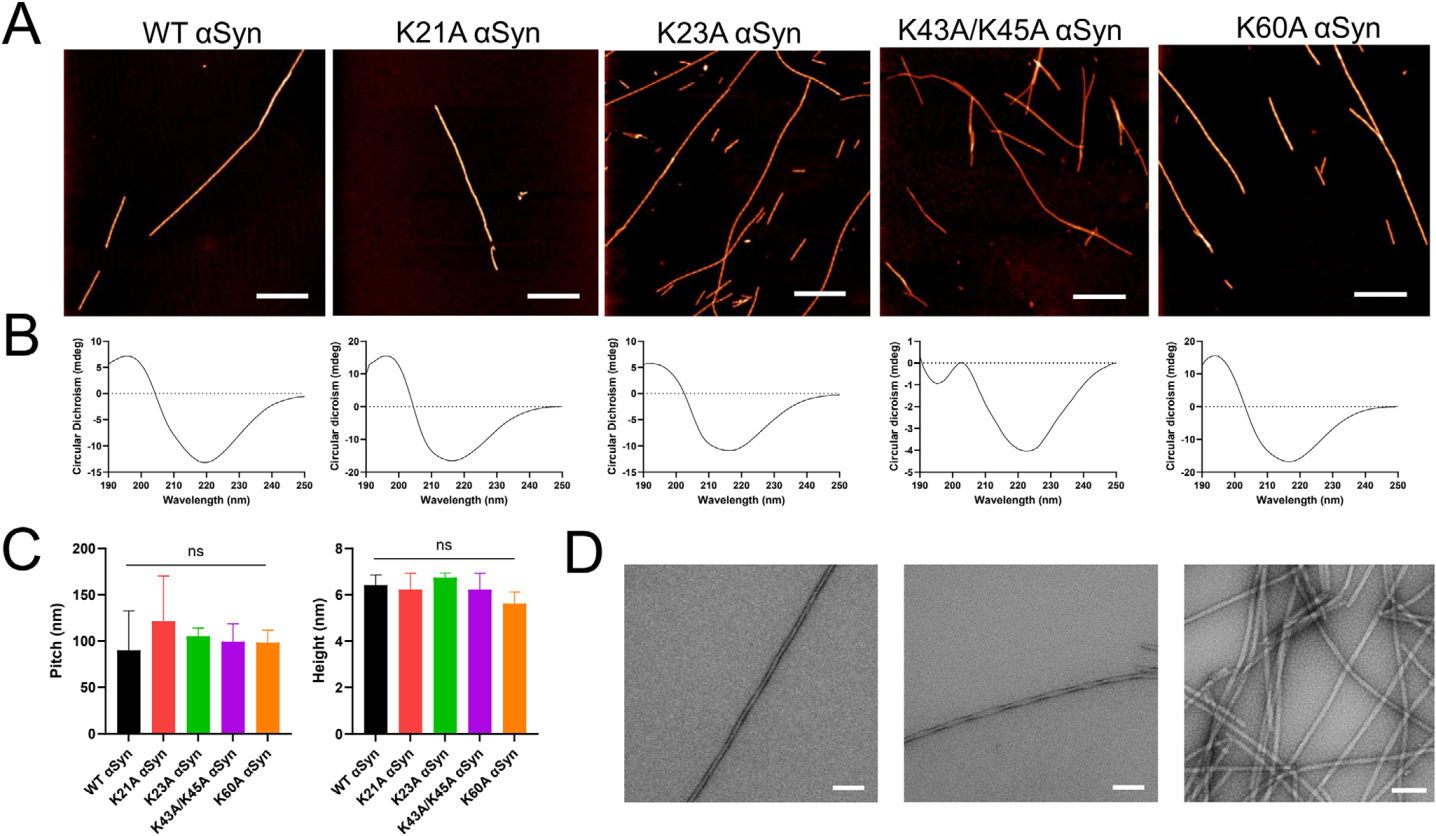
Characterization of variant αSyn amyloids by AFM, CD, and TEM. A) AFM images of amyloids, scale bar 1 µm. B) Secondary structure analysis of 10 µm amyloids by CD spectroscopy. C) Periodicity (left) and cross section (right) of fibers. Statistical analysis performed by ANOVA, ns (not significant). D) Negative Stain TEM images of 50 µm WT (left), K43A/K45A (middle), and K23A (right) αSyn amyloids. Scale bar 100 nm.

To address the role of the lysines in the cavity and in the folded N‐terminal part, αSyn variants K60A, K43A/K45A (double mutant), K21A, and K23A were prepared. All variants formed amyloids as evident by atomic force microscopy (AFM) (**Figure** **3**A) and circular dichroism (CD) spectroscopy (Figure 3B). Far‐UV CD confirmed the presence of β‐sheet structure for all species (Figure 3B), and based on the AFM, all amyloids had a periodicity along the fiber axis of ≈100 nm (Figure 3C, left) and a cross section of around 6 nm (Figure 3C, right). Consistent with the AFM data, negative‐stain transmission electron microscopy (TEM) images showed similar amyloids for WT, K23A, and K43A/K45A αSyn (Figure 3D) (K21A, K60A; data not shown).

**Figure 4:**
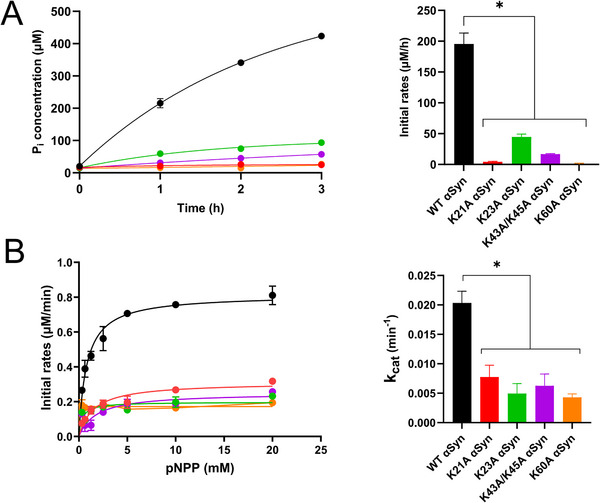
Catalytic activity of αSyn amyloids. A) ATP hydrolysis of αSyn amyloid variants: Concentration of free phosphate released after ATPase activity of 40 µm αSyn amyloids at different time points, WT (black), K21A (red), K23A (green), K43A/K45A (purple), K60A (orange) in the presence of 1mM ATP (left). Initial rates of ATP hydrolysis as a function of different αSyn amyloids (right). Error bars, mean ± SD (*N* = 3). Statistical analysis performed by ANOVA, ^*^ *p*<0.05. B) Dephosphorylation of pNPP by αSyn amyloid variants: Michaelis–Menten plots of pNPP hydrolysis for WT (black) and variant αSyn amyloids (K21A red, K23A green, K43A/K45A purple, K60A orange) (left). k_cat_ values from Michaelis–Menten plots for the αSyn amyloid variants (right). Error bars, mean ± SD (*N* = 3). Statistical analysis performed by ANOVA, ^*^ *p*<0.05.

Since the collection of low‐resolution data showed the mutant amyloids to be similar in terms of overall fold, we investigated whether these αSyn variant amyloids mediated ATP hydrolysis using the Malachite Green assay with 1 mm ATP (**Figure** **4**). Whereas we detect over 400 µm free phosphate after 3 h of incubation with WT αSyn amyloids (Figures 1D and 4A), the mutant amyloids showed little to no phosphate generation during this time interval (Figure 4A).

Since we initially found that ATP blocked pNPP activity of WT αSyn amyloids, suggesting overlapping binding sites, we also tested pNPP activity with the mutated amyloids. In agreement with the ATP hydrolysis data, we find that pNPP hydrolysis is impaired in mutant amyloids as well (Figure 4B). The Michaelis–Menten plots depicted in Figure 4B clearly show that mutated amyloids exhibit much lower k_cat_ values for pNPP hydrolysis (between 0.008‐0.004 min^−1^) as compared to the value for WT αSyn amyloids (0.02 min^−1^). Thus, the lysine residues appear important for phosphoester bond cleavage in both ATP and pNPP.

### 2.4 Structure of a Mutant Amyloid Lacks Extra β‐Strand

**Figure 5:**
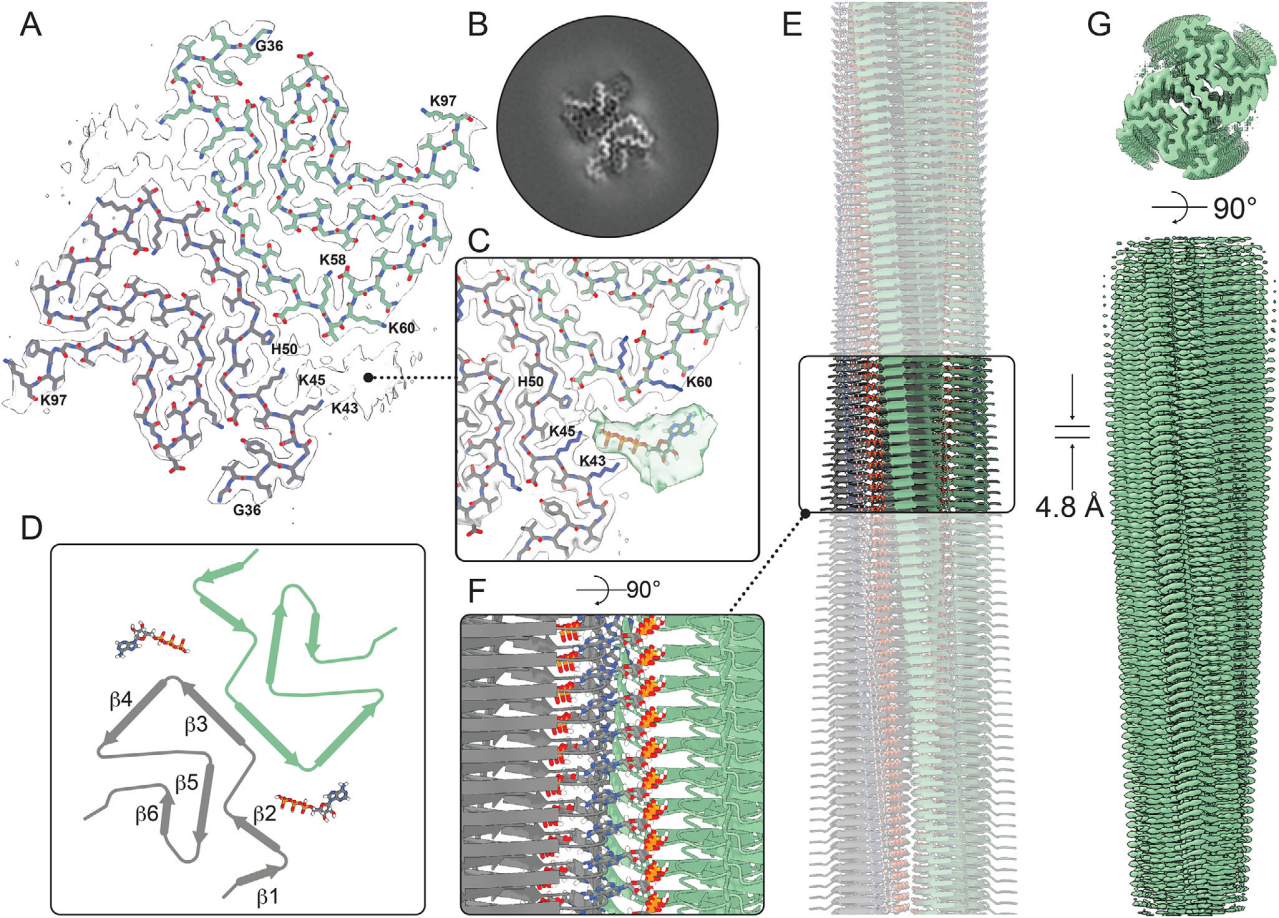
CryoEM structure of K21A αSyn amyloids in the presence of ATP. A) Post‐processed cryo‐EM density map overlayed with the atomic model. In the post‐processed map, there is a low‐resolution cryo‐EM density observable in the vicinity of K43 and K45 of the ATP molecule. B) Cryo‐EM density map from the 3D refinement of K21A αSyn. C) Chimeric cryo‐EM density map combining the post‐processed map (grey) with the 3D refinement map (green). ATP was modeled into the green density using ChimeraX. The binding of ATP reorients the N‐terminus of the K21A αSyn (i.e., residues N‐terminal of K43) such that they point away from the ATP cavity (in contrast to observed in the WT αSyn‐ATP structure). D) Schematic representation of the type 1A K21A αSyn polymorph bound to ATP, illustrating the overall polymorphism of the fibril. E) (F, zoom in) Representative illustration of ATP binding on the surface of the amyloid fibril. G) Post‐processed cryo‐EM density map of the ATP‐bound fibril, used to build the atomic mode.

To assure that the overall fold of the lysine‐mutated αSyn amyloids had not changed, we solved the cryo‐EM structure of the K21A αSyn amyloid in the presence of ATP to 3.13 Å resolution (**Figure** **5**; Table S1 and Figure S4, Supporting Information). The structure reveals a type‐1A fold but without the extra N‐terminal β‐strand comprising residues 16‐22. Still, we detected density for ATP, in the same cavity as found with the WT αSyn amyloids, implying that ATP binds but is not hydrolyzed. In accord, the ATP molecule is found in a different orientation in the K21A amyloid as compared to in the WT αSyn amyloid (compare Figures 2C and 5C). In the K21A amyloid, the phosphates of ATP point inward toward His50, whereas in the WT αSyn structure, the ATP phosphate tail points outward toward the new β‐strand of N‐terminal residues. Thus, the orientation of ATP in the WT αSyn amyloid, along with the extra β‐strand that encloses the cavity, appears essential feature of the system to promote chemical reactivity. Interestingly, in the ATP‐K21A structure, there is a small rearrangement (with respect to the ‘normal’ type 1A‐fold) of the N‐terminal stretch (residues 42‐38) in the direction away from the cavity/ATP site (Figure 5A–C). This is the opposite direction to the ‘fold‐over’/enclosing rearrangement of the N‐terminus observed in the WT αSyn‐ATP complex.

The fact that ATP binds to the K21A amyloids, despite a lack of chemical reactivity, was independently confirmed in solution using the fluorescent analog MANT‐ATP, which has an environment‐sensitive fluorophore attached to the ribose moiety (Figure S5, Supporting Information). For both WT and K21A amyloids, ATP binding was detected in the micromolar range (estimated ATP dissociation constants around 30–40 µm at these conditions)

### 2.5 Computational Support for Divergent ATP Orientations in WT and Mutant Amyloids

To further assess the ATP interaction with WT and mutant amyloid structures, we performed molecular docking followed by molecular dynamics (MD) simulations.^[^ ^25^ ^]^ As we have Mg^2+^ in the solution, we decided to use Mg^2+^‐ATP for the docking. The binding mode observed from the docking of Mg^2+^‐ATP into the closed cavity of the WT structure and the open cavity of the K21A αSyn mutant agreed with the distinct ATP orientations observed in each cryo‐EM structure (Figure S6, Supporting Information). Subsequent MD simulations of the complexes for 100 ns revealed that, in each case, the ATP interaction was stable over time, although ATP displayed local dynamics (Figure S7, Supporting Information).

## 3 Discussion

We here show that polymorph type 1A of αSyn amyloids bind ATP in a specific lysine‐rich cavity positioned between the two protofilaments, with the formation of a cavity‐enclosing βstrand not typically observed in type 1A αSyn structures. The interaction results in ATP dephosphorylation and is dependent on the presence of several lysine residues positioned in the cavity. The observed ATP binding cavity matches what we recently found to be the in silico favored site for pNPP in type 1A αSyn amyloids.^[^ ^25^ ^]^ Although amyloids of Lys mutants of αSyn cannot hydrolyze ATP, they still bind the ligand in the identified cavity, but (at least for K21A) in a different orientation.

### 3.1 ATP Binding Modulates the Polymorph Structure Type 1A

As expected, the overall fold of the polymorph type 1A of αSyn amyloids does not alter upon addition of ATP. However, the addition of ATP modulates the fold by inducing the formation of an additional N‐terminal β‐strand comprising residues 16‐22. With this rearrangement, critical Lys residues form a positively charged and enclosed cavity for the negatively charged ATP. This binding mode can be considered to follow an induced fit mechanism as the ligand induces an altered structure. This is possible because residues 16‐22 are not part of the rigid core structure of the amyloid. The additional β‐strand in the N‐terminus of one protofilament interacts, in the same vertical layer of the dimeric amyloid structure, with the ordered core of the other protofilament, resulting in a peptide loop enclosing the protofilament interface cavity and adding a β‐sheet to the overall cross‐β structure. Structural rearrangements upon ligand binding are often observed for evolutionary‐optimized enzymes, placing the protein in a reactive state, and may also be the case here. It is intriguing to note that the rather stable and repetitive structure of an amyloid, often considered an inert rock‐like entity, has the capacity to adapt its structure semi‐locally due to small‐molecule binding.

### 3.2 On the Potential ATP Hydrolysis Mechanism

As stated earlier, ATP is the primary energy currency in biological systems, and its hydrolysis drives most metabolic and biosynthetic reactions in cells. There are a plethora of structural motifs used by the proteins to bind and hydrolyze ATP.^[^ ^26^ ^]^ Often positively charged Lys or (sometimes Arg) side chains interact with both the negatively charged phosphates and the sugar OH moieties of ATP. In addition, positively charged ions, such as Mg^2+^ ions, often interact with the ATP terminal γ‐phosphate. In the prominent Walker A motif, a Lys side chain interacts with both the β‐ and γ‐phosphates, with the latter also interacting with a Mg^2+^ ion, while another Lys residue coordinates with the sugar OH moieties of the ATP.^[^ ^27^ ^]^

In contrast to evolved ATP catalytic sites, the binding pocket in the αSyn amyloids has not been optimized, as αSyn amyloid formation is associated with disease. This is also reflected by the apparent low catalytic rate for the amyloids (k_cat_ of 0.08 min^−1^ at 1 mm ATP; however, this value is a lower limit as we assume one site per polypeptide, but likely they span more than one peptide layer), which is lower than ATP hydrolysis rates of ‘normal’ ATP hydrolyzing proteins. For example, ATP hydrolysis rates by cpn60 and Hsp90 chaperones have been reported in the range of 0.8 min^−1^,^[^ ^28^ ^]^ the copper transporter ATP7B shows an ATP hydrolysis rate of 3 min^−1^,^[^ ^29^ ^]^ whereas microtubule‐stimulated ATP hydrolysis by kinesin dimers can reach rates over 2000 min^−1^.^[^ ^30^ ^]^ The lack of a well‐defined ATP density in the cryo‐EM data analysis further hints at a non‐optimized active site in the amyloid. Nonetheless, based on the indicated ATP orientation in the amyloid structure in Figure 2, Lys side chains interact with both the sugar of the nucleotide (Lys43 and Lys45) as well as the phosphates (Lys60 and Lys21), which is similar to evolved ATP‐binding sites. The Lys residues (K43, K45, K60, K21, K23) are all crucial for facilitating hydrolysis, as we observe loss of activity for all our K to A mutants (Figure 4).

Still, ATP binding is possible at least in the case of the K21A αSyn variant (Figure 5). In the structure of the inactive K21A αSyn variant, as well as in our in silico docking, ATP is found in a different orientation, and there is no additional β‐strand for residues 16‐22. It is therefore reasonable to propose that the active site (for ATP hydrolysis) involves K43 and K45 anchoring the adenine‐sugar and K60 and K21 (and possibly K23) interacting with, and activating, the phosphate groups, with the latter located in the recruited, additional β‐strand. Mg^2+^ ions appear to play no prominent role since the catalytic activity of the WT αSyn amyloids was not much altered in their absence (Figure S8, Supporting Information).

The following plausible mechanism of catalytic action emerges: With an induced fit mechanism, ATP binding to type 1A αSyn amyloids (anchoring at K43 and K45) recruits an additional β‐strand comprising residues 16‐22. This engagement orchestrates the formation of a catalytically‐active site with K60 and K21 positioned near the phosphate groups and, due to the enclosure of the cavity, restricts bulk water access, potentially along with one or several constrained water molecules inside the cavity, which may enhance reactivity. The β‐strand formation can thus be regarded as a shielding lid over the cavity. In the ATP‐αSyn amyloid complex depicted in Figure 2, the distances between K60 and K21 to the β‐phosphate and γ‐phosphate are, however, suboptimal (i.e., too large) when compared with corresponding ATP motifs in other ATPases (e.g.,^[^ ^31^ ^]^). Thus, the ATP‐αSyn complex may be in a high‐energy, reactive state: when the hydrolysis reaction is completed (cleaving off one or two phosphate groups), the β‐strand is released (returning to low‐energy), and product molecules are released. This makes the amyloid system ready to restart catalysis again by binding a new ATP.

### 3.3 Catalytic Activity of Amyloids

Catalytic amyloids with a diverse set of activities in material sciences,^[^ ^14b,c^ ^]^ in the origin of life,^[^ ^14^, ^32^ ^]^ as well as for pathological amyloids have been documented recently.^[^ ^11^, ^13^ ^]^ Here, we demonstrate via detection of free inorganic phosphate that type 1A αSyn amyloids hydrolyze ATP. We note that a recent preprint posted on bioRxiv^[^ ^33^ ^]^ also reports ATP hydrolysis by WT α‐syn amyloids, although no mechanistic or structural analysis was performed. In that study, HPLC was used to show sequential degradation products that included ADP, AMP, and an unknown species.

Previous work on synthetic, designed peptides^[^ ^34^ ^]^ has shown the capacity of peptide‐based amyloids (induced to form amyloids by Mn^2+^ addition) to promote ATP hydrolysis. However, the catalytic rate was around two orders of magnitude smaller than the one demonstrated here for αSyn amyloids. As the designed peptide amyloids lacked Lys and Arg residues, the positively charged N‐terminus was proposed to be involved in reactivity. We hypothesize that the large difference in catalytic activity between the synthetic peptide and αSyn amyloids is due to a) the presence of the Lys residues in the latter that enhance the affinity for ATP, as shown for small RNA molecules,^[^ ^35^ ^]^ and b) the structural recruitment of the additional β‐strand in the αSyn amyloids that by enclosing the cavity provides a water‐confined environment, reminiscent of enzyme active sites.

### 3.4 Could There be a Biological Relevance for the ATP Hydrolysis?

With several mm concentration of ATP in cells, an amazing ATP production rate in humans (producing our own weight of ATP every day), the slow rate we report here (k_cat_ of 0.08 min^−1^; estimated catalytic efficiency around 30 m ^−1^s^−1^ when using MANT‐ATP affinity as K_M_), along with the particular αSyn polymorph studied here not detected in patients (yet),^[^ ^36^ ^]^ the biological relevance of the presented amyloid‐mediated ATP hydrolysis may appear enigmatic. Still, the available free ATP concentration may vary locally in cells, and dopaminergic neurons are particularly sensitive to ATP levels due to their constant dopamine production and release. It has been reported that cytoplasmic ATP levels decrease early in PD, preceding neuronal cell death,^[^ ^21^, ^37^ ^]^ and amyloid‐mediated disruption of mitochondrial membranes may contribute to this.^[^ ^22^ ^]^ The high concentration of αSyn amyloid fibrils in a Lewy body, and the locally high concentration of active sites within each fibril due to the repetitive cross‐β nature, at the sub‐nm level, may result in depletion of ATP at amyloid surfaces and in Lewy bodies in vivo, if the amyloids have a polymorph that displays catalytic activity.

This might be of particular relevance for ATP‐dependent chaperones, such as Hsp70, that may dissociate amyloids in an ATP‐dependent manner.^[^ ^38^ ^]^ While Hsp70 has a low ATP hydrolysis rate by itself, between 0.02 and 1.0 min^−1^, this rate can be increased up to 1000‐fold in the presence of co‐chaperones and substrates.^[^ ^39^ ^]^ Although chaperones, when activated, may hydrolyze ATP faster than of αSyn amyloids, the local concentration of active sites on αSyn amyloids (up to two per 4.8 Å, which is 4000 sites for an amyloid of 1 µm length) may be several orders of magnitude higher than the local concentration of chaperones and co‐chaperones near the amyloids. In other words, the chaperones may enter an ATP‐depleted zone when approaching amyloids in vivo, limiting their rescue activities.

## 4 Conclusion

In summary, three important conclusions emerge from this study. First, WT αSyn type 1A amyloids bind and hydrolyze ATP in a specific lysine‐rich cavity positioned between the two protofilaments. The presence of amyloid‐mediated ATP hydrolysis in vivo will depend on each amyloid's polymorph (as it governs the chemical features of the surface); when activated, it may contribute to disease progression by reducing (local) energy supply in the cell. Second, when ATP binds to αSyn amyloids, the amyloid rearranges such that an additional β‐strand is formed in a disordered part of the peptide. This observation demonstrates that amyloid structures (and thus their exposed surfaces) can be modulated by small molecules after amyloid formation. This opens the possibility to silence amyloid toxicity (such as secondary nucleation, cell‐to‐cell spreading) via small‐molecule‐induced ‘deactivation’ of amyloid surfaces. Third, although the ATP hydrolysis rate detected here is low, the high local density of active sites on amyloid fibrils may result in ATP‐depleted zones close to amyloid surfaces. This may abolish energy‐consuming activities, such as ATP‐dependent chaperone‐mediated disassembly. This will, in essence, render the amyloids ‘invisible’ to rescue and/or degradation mechanisms and is an entirely new chemical concept in the field of amyloid diseases.

## Conflict of Interest

The authors declare no conflict of interest.

## Supporting information



Supporting Information

## Data Availability

The data that support the findings of this study are available in the supplementary material of this article.

## References

[1] F. Chiti , C. M. Dobson , Annu. Rev. Biochem. 2017, 86, 27. 28498720 10.1146/annurev-biochem-061516-045115

[2] M. R. Sawaya , M. P. Hughes , J. A. Rodriguez , R. Riek , D. S. Eisenberg , Cell 2021, 184, 4857. 34534463 10.1016/j.cell.2021.08.013 PMC8772536

[3] a) M. L. Evans , M. R. Chapman , Biochim. Biophys. Acta 2014, 1843, 1551; 24080089 10.1016/j.bbamcr.2013.09.010 PMC4243835

[4] a) A. L. Fink , Acc. Chem. Res. 2006, 39, 628; 16981679 10.1021/ar050073t

[5] E. P. De Mattos , A. Wentink , C. Nussbaum‐Krammer , C. Hansen , S. Bergink , R. Melki , H. H. Kampinga , J. Parkinson's Dis. 2020, 10, 369. 31985474 10.3233/JPD-191790 PMC7242842

[6] a) A. John , W. van der Pluijm , Ann. Neurol. 2018, 84, S219;

[7] a) M. S. Goldberg , P. T. Lansbury , Nat. Cell Biol. 2000, 2, E115; 10878819 10.1038/35017124

[8] M. H. Polymeropoulos , C. Lavedan , E. Leroy , S. E. Ide , A. Dehejia , A. Dutra , B. Pike , H. Root , J. Rubenstein , R. Boyer , E. S. Stenroos , S. Chandrasekharappa , A. Athanassiadou , T. Papapetropoulos , W. G. Johnson , A. M. Lazzarini , R. C. Duvoisin , G. Di Iorio , L. I. Golbe , R. L. Nussbaum , Science 1997, 276, 2045. 9197268 10.1126/science.276.5321.2045

[9] a) J. Xu , S. Y. Kao , F. J. Lee , W. Song , L. W. Jin , B. A. Yankner , Nat. Med. 2002, 8, 600; 12042811 10.1038/nm0602-600

[10] a) W. Peelaerts , L. Bousset , A. Van der Perren , A. Moskalyuk , R. Pulizzi , M. Giugliano , C. Van den Haute , R. Melki , V. Baekelandt , Nature 2015, 522, 340; 26061766 10.1038/nature14547

[11] a) P. Wittung‐Stafshede , Biochem. Soc. Trans. 2023, 51, 1967; 37743793 10.1042/BST20230617 PMC10657172

[12] I. Horvath , K. A. Mohamed , R. Kumar , P. Wittung‐Stafshede , Int. J. Mol. Sci. 2023, 24, 12849. 37629028 10.3390/ijms241612849 PMC10454467

[13] a) E. Arad , A. Baruch Leshem , H. Rapaport , R. Jelinek , Chem Catal. 2021, 1, 908;

[14] a) J. Greenwald , M. P. Friedmann , R. Riek , Angew. Chem. Int. Ed. 2016, 55, 11609; 10.1002/anie.201605321 27511635

[15] a) K. Jiang , S. Rocha , R. Kumar , F. Westerlund , P. Wittung‐Stafshede , Biochem. Biophys. Res. Commun. 2021, 568, 43; 34175689 10.1016/j.bbrc.2021.06.059

[16] I. Horvath , O. A. Aning , S. Kk , N. Rehnberg , S. Chawla , M. Molin , F. Westerlund , P. Wittung‐Stafshede , ACS Chem. Neurosci. 2025, 16, 355. 39782739 10.1021/acschemneuro.4c00461 PMC11803820

[17] G. W. Davis , Neuron 2020, 105, 591. 32078791 10.1016/j.neuron.2020.01.024

[18] O. Brylski , P. Shrestha , P. Gnutt , D. Gnutt , J. W. Mueller , S. Ebbinghaus , Front.Mol. Biosci. 2021, 8, 790304. 34966785 10.3389/fmolb.2021.790304 PMC8710738

[19] J. V. Greiner , T. Glonek , Biology 2021, 10, 1166. 34827159 10.3390/biology10111166 PMC8615055

[20] M. Nakano , H. Imamura , N. Sasaoka , M. Yamamoto , N. Uemura , T. Shudo , T. Fuchigami , R. Takahashi , A. Kakizuka , EBioMedicine 2017, 22, 225. 28780078 10.1016/j.ebiom.2017.07.024 PMC5552266

[21] C. Zhang , R. A. Rissman , J. Feng , J. Alzheimers Dis. 2015, 44, 375. 25261448 10.3233/JAD-141890 PMC4305018

[22] O. Lurette , R. Martín‐Jiménez , M. Khan , R. Sheta , S. Jean , M. Schofield , M. Teixeira , R. Rodriguez‐Aller , I. Perron , A. Oueslati , E. Hebert‐Chatelain , Cell Death Dis. 2023, 14, 729. 37949858 10.1038/s41419-023-06251-8 PMC10638290

[23] E. R. Kamski‐Hennekam , J. Huang , R. Ahmed , G. Melacini , Chem. Sci. 2023, 14, 9933. 37736631 10.1039/d3sc03612j PMC10510630

[24] S. Ley‐Ngardigal , G. Bertolin , FEBS J. 2022, 289, 7940. 34437768 10.1111/febs.16169

[25] S. Parate , F. Buratti , L. A. Eriksson , P. Wittung‐Stafshede , Biophys. J. 2025, 124, 2418. 40538035 10.1016/j.bpj.2025.06.017 PMC12414660

[26] M. Rappas , H. Niwa , X. Zhang , Curr. Protein Pept. Sci. 2004, 5, 89. 15078220 10.2174/1389203043486874

[27] C. Ramakrishnan , V. S. Dani , T. Ramasarma , Protein Eng. 2002, 15, 783. 12468712 10.1093/protein/15.10.783

[28] a) J. C. Young , F. U. Hartl , Embo J. 2000, 19, 5930; 11060043 10.1093/emboj/19.21.5930 PMC305790

[29] R. M. Bitter , S. Oh , Z. Deng , S. Rahman , R. K. Hite , P. Yuan , Sci. Adv. 2022, 8, abl5508. 10.1126/sciadv.abl5508 PMC8896786 35245129

[30] D. D. Hackney , J. Biol. Chem. 1994, 269, 16508. 8206961

[31] M. M. Ali , S. M. Roe , C. K. Vaughan , P. Meyer , B. Panaretou , P. W. Piper , C. Prodromou , L. H. Pearl , Nature 2006, 440, 1013. 16625188 10.1038/nature04716 PMC5703407

[32] a) S. K. Rout , D. Rhyner , R. Riek , J. Greenwald , Chem. Eur. J. 2022, 28, 202103841; 10.1002/chem.202103841 PMC9299922 34812556

[33] C. Castillo‐Caceres , E. Nova , R. Diaz‐Espinoza , bioRxiv 2025, 20250403, 647058.

[34] C. Castillo‐Caceres , E. Duran‐Meza , E. Nova , R. Araya‐Secchi , O. Monasterio , R. Diaz‐Espinoza , Biochim. Biophys. Acta 2021, 1865, 129729. 10.1016/j.bbagen.2020.129729 32916204

[35] S. K. Rout , R. Cadalbert , N. Schröder , J. Wang , J. Zehnder , O. Gampp , T. Wiegand , P. Güntert , D. Klingler , C. Kreutz , A. Knörlein , J. Hall , J. Greenwald , R. Riek , J. Am. Chem. Soc. 2023, 145, 21915. 37782045 10.1021/jacs.3c06287 PMC10571083

[36] L. Frey , D. Ghosh , B. M. Qureshi , D. Rhyner , R. Guerrero‐Ferreira , A. Pokharna , W. Kwiatkowski , T. Serdiuk , P. Picotti , R. Riek , J. Greenwald , eLife 2024, 12, RP93562. 39196271 10.7554/eLife.93562 PMC11357353

[37] L. Y. Shields , B. A. Mendelsohn , K. Nakamura , in Techniques to Investigate Mitochondrial Function in Neurons (Eds: S. Strack , Y. M. Usachev ), Humana Press, New York, USA 2017.

[38] X. Gao , M. Carroni , C. Nussbaum‐Krammer , A. Mogk , N. B. Nillegoda , A. Szlachcic , D. L. Guilbride , H. R. Saibil , M. P. Mayer , B. Bukau , Mol. Cell 2015, 59, 781. 26300264 10.1016/j.molcel.2015.07.012 PMC5072489

[39] M. P. Mayer , B. Bukau , Cell. Mol. Life Sci. 2005, 62, 670. 15770419 10.1007/s00018-004-4464-6 PMC2773841

