# Supplementary material for: ATP Hydrolysis by α‐Synuclein Amyloids is Mediated by Enclosing β‐Strand
Source: Adv Sci (Weinh). 2025 Oct 16;12(44):e08441. doi: 10.1002/advs.202508441 (PMC12667531; doi:10.1002/advs.202508441)
Supplement: Supplementary file 1 — Supporting Information [file ADVS-12-e08441-s001.pdf]

Supporting Information

ATP HYDROLYSIS BY α-SYNUCLEIN AMYLOIDS IS MEDIATED BY ENCLOSING β-STRAND

Lukas Frey, Fiamma Ayelen Buratti, Istvan Horvath, Shraddha Parate, Ranjeet Kumar, Roland Riek, Pernilla Wittung-Stafshede

List of content:

Experimental Section

Table S1

Figures S1-S8

References for experimental section

Experimental Section

Protein expression and purification

pET-21a (+) vectors harboring the genes of WT αSyn, three point-mutated variants (K21A, K23A, K60A; in which lysines at positions 21, 23 and 60 had been exchanged with alanine one at a time), and one double mutant (K43A/K45A where the lysines at positions 43 and 45 had both been exchanged with alanine) were transformed into BL21 (DE3) (Novagen) cells. Transformants were first grown to an OD_{600} of 0.6 in LB containing 100 μg/mL carbenicillin at 37 ºC, then induced with 1 mM isopropyl b-D-1-thiogalactopyranoside (IPTG) and grown overnight at 20 ºC. Cells were lysed by sonication in pulse mode in 20 mM Tris–HCl buffer pH 8.0 in the presence of protease inhibitor cocktail (Roche) in an ice bath. After sonication, the lysate was treated with a universal nuclease (Pierce) for 15 min at room temperature. The lysate was then heated at 90 ºC for 10 min followed by centrifugation for 30 min at 15,000 g. The centrifuged lysate after filtration (Nalgene rapid-flow filter, 0.2 μm PES membrane; Thermo Fisher Scientific) was loaded on a pre-equilibrated 5 mL HiTrap Q FF anion exchange column (Cytiva) and eluted by a linear gradient of 1 M NaCl in 20 mM Tris–HCl, pH 8.0. Fractions contained the protein were combined and concentrated with Ultra-15 Ultracel 10 K centrifugal filtration devices (Millipore). The concentrate was then loaded on to HiLoad 16/600 Superdex 75 pg column (Cytiva) and retrieved in 20 mM Tris-sulfate buffer, pH 7.4. Purity was confirmed by a single-band on SDS-PAGE gel and a single elution peak in SEC. Protein samples were flash frozen and stored at -80 ºC until use. To determine protein concentration of WT and variant αSyn, the extinction coefficient of 5960 M^{-1} cm^{-1} at 280 nm was used.

Preparation of amyloids

Amyloid samples for biochemical and structural experiments were in seeded reactions of pre-made amyloids mixed with fresh monomers. Amyloid seeds were prepared from 100 μM αSyn incubated with agitation using glass beads at 37 ºC in TBS (TRIS Buffered Saline; 50 mM TRIS and 150 mM NaCl at pH 7.4) in a plate reader incubator for 3 days. The resulting samples were aliquoted, flash frozen with liquid nitrogen, stored at -80 ºC and used as premade seeds. To create final samples of amyloids, pre-made seeds (12.5 μM in monomer units) were mixed with 250 μM fresh monomeric α-synuclein. The mixture was incubated in thermomixer for 4 days at 37 ºC. At the end of the incubation, samples were centrifuged at 13500*g* for 30 min to separate amyloid fibers from remaining monomers and smaller assemblies. The pellet was resuspended in TBS, and the protein concentration of the pellet fraction was approximated from the protein concentration in the supernatant measured by absorbance at 280 nm.

pNPP assay

para-Nitrophenyl-ortophosphate (pNPP) (Sigma-Aldrich) was dissolved in Milli-Q water at a concentration of 50 mM. The reaction buffer was 20 mM Tris Buffer pH 7.6, 5 mM MgCl2, 1 mM EGTA. The protein solutions were diluted into water and the required amount of pNPP was added to the protein (monomeric or amyloid fiber forms of αSyn variants) solution. Blank measurements were performed by omitting the protein from samples but using the same volume of water mixed into the buffer. The corresponding blank measurements were subtracted from the absorbance values obtained in the presence of added protein. The samples were incubated in 96-well, half area transparent-bottom plates with a nonbinding surface (CLS3881; Corning, Corning, NY) at 37 ºC using a plate reader incubator instrument (Fluorostar Optima; BMG Labtech, Ortenberg, Germany). Absorbance at 410 nm was measured every 3 min over 80 min. The initial rate calculation was performed on data points between 10 and 40 min. For the calculation of initial rates, an extinction coefficient of 13 500 M^{-1} cm^{-1} for pNP at 410 nm was used.

ATP assay

Adenosine 5'-triphosphate (ATP) (Sigma-Aldrich) was dissolved in Milli-Q water at a concentration of 20 mM. The reaction buffer is the same used for phosphatase assay. The

protein solutions were diluted into water and 1mM ATP was added to the protein (40 μM monomeric or 40 μM amyloid fiber forms of αS variants) solution. The samples were incubated in the thermomixer at 37 ºC with 500 rpm shaking. Samples at different time points were stopped freezing them at -20 ºC. Once, all the samples are collected, were thawed, and 20 μl sample were mixed with 40 μl Malachite green (MAK308-1KT, Sigma-Aldrich), in a plate of 96-well, half area transparent-bottom plates with a nonbinding surface (CLS3881; Corning, Corning, NY). The reaction was incubated for 30 minutes at room temperature for color development, then absorbance was measured at 610 nm on a plate reader. Standards phosphate concentrations were prepared to calculate the exact concentration of our samples.

Aggregation kinetic assay

αSyn monomers (50 μM) were mixed with 5 μM of pre-made amyloid seeds in the presence of 25 μM of ThT in TBS buffer pH 7.6. One glass bead was added per well. The reaction was followed over time by thioflavin-T (ThT) fluorescence in a FLUOstar Omega (BMG Labtech) reader at 37 ºC using cycles of 5 min shaking (300 rpm) followed by 5 min rest intervals.

Proteinase K digestion

Digestion of 50 μM of fibrils was performed with 3.8 μg/ml of Proteinase K (AM2546, Ambion) for different times at 37 ºC. The reaction was stopped by loading buffer and boiled at 95 ºC for 5 minutes, followed by loading into a NuPAGE 4-12% Bis Tris gel (NP0322, Invitrogen).

Fluorescent ATP for binding to amyloids

To probe ATP binding to amyloids, the nucleotide analog MANT-ATP (N-Methylanthraniloyl)-ATP (M12417, Invitrogen) with a environment-sensitive fluorophore attached to the ribose moiety was used. Different concentrations of αSyn amyloids were mixed with 20 μM MANT-ATP in 20 mM Tris Buffer pH 7.6, 5 mM MgCl2, 1 mM EGTA. Fluorescence was observed by FLUOstar Omega (BMG Labtech) reader. For emission spectra, 20 μM MANT-ATP samples with and without 80 μM amyloid fibrils were excited at 355 nm in a fluorimeter (Varian Cary, Eclipse).

Atomic Force Microscopy

Amyloid fibers were diluted until reach 10 μM into Milli-Q water and deposited on freshly cleaved mica. After 30 min, the mica was rinsed with filtered Milli-Q water and dried under a gentle nitrogen stream. Images were recorded on an NTEGRA Prima setup (NT-MDT, Moscow, Russia) using a gold coated single-crystal silicon cantilever (NT-MDT, NSG01, spring constant of ~5.1 N/m) and a resonance frequency of ~180 kHz in tapping mode. 512 × 512-pixel images were acquired with a scan rate of 0.5 Hz. Images were analyzed using the WSxM 5.0 software.

Circular dichroism (CD)

For CD measurement, 300 μl of the sample was transferred to a 0.1 cm quartz cuvette. CD data were collected from 190 to 250 nm by using Chirascan spectrophotometer (Applied Photophysics) at 20°C, 1 time-per-point (s) in 1 nm steps. The datasets were averaged from three repeats. All spectra were baseline corrected against buffer in MilliQ water.

Negative stain transmission electron microscopy (TEM)

Amyloid fibers were diluted until reach 30 μM into Milli-Q water and 5 μl of the sample was incubated on grids for 2 minutes. After blotting excess sample with filter paper, it was washed 2 times with water droplets and then stained with 2% uranyl acetate for 45 sec. Grids were blotted and dry for 10 min at room temperature (TALOS L120C, Thermo Scientific).

Cryo-EM

Quantifoil copper 300 mesh 2/1 grids were glow discharged using -25 mA for 45s prior freezing. The αSyn fibrils were diluted to 60 μM αSyn concentration with TBS, supplemented with 1 mM ATP, 1 mM $MgCl_2$. For the control samples ATP and $MgCl_2$ were not supplemented. The 4 μl of samples were vitrified with a Vitrobot Mark IV (Thermo Fisher) using a blot time

of 5s, blot force 1, waiting time of 20 s, temperature of 15°C and 100% humidity. Cryo-EM data was collected on a Titan Krios 3Gi or 4 using a magnification of 130 kx, Gatan K3 camera, Gatan energy filter with slit width of 20 eV, a total dose of 53-63 e/Å^2 (see **Table S1**) using 7.2-7.8 e/pixel/s at a pixel size 0.65 Å in counted super resolution mode.

Cryo-EM data was processed using the Relion 5 software and the helical refinement procedure developed by Scheres and coworkers[1]. For motion correction the Relion's own implementation was used and the contrast transfer function (CTF) was estimated using CTFFIND5[2]. For filament selection, crYOLO or Topaz was used [3] and segments were extracted with a 333 Å box at a pixel size of 2.6 Å for a first 2D classification and later reextracted at a pixel size of 1.3 Å followed by another 2D classification round. For 3D refinement the initial model was generated with *relion_helix_inimodel2d* [4] followed by subsequent 3D refinement, Bayesian polishing, CTF-refinement steps to reach a final resolution of 3.08, 3.20 and 3.13 Å for WT αSyn + ATP, WT αSyn, and K21A variant + ATP, respectively.

In silico ATP docking and MD simulations

The WT αSyn cryo-EM structure includes the N-terminal β-strand (residues 16-22) and the core region (residues 36-98) with three chains per protofilament (six chains in total as two protofilaments). The missing segment (residues 23-35) was modelled using the *Protein Linker Design* module within Schrödinger Maestro (Schrödinger 2024–4, www.schrodinger.com) to generate a structurally plausible linker connecting the two regions. The K21A αSyn mutant cryo-EM structure with five chains per protofilament (ten chains in total as two protofilaments) include a continuous peptide and could be directly used. The WT and mutant models were refined using the *Protein Preparation Wizard*, followed by a 100 ns molecular dynamics (MD) simulation in *Desmond* under NPT conditions (300 K, 1 atm) to equilibrate the structures prior to docking [5]. ATP was prepared in its Mg^{2+}-coordinated form to reflect experimental conditions using *LigPrep* with prior ionization states at pH 7.4 [6]. The most common ATP-bound conformation in biological systems involve coordination of Mg^{2+} with the oxy-anion of the β- and γ-phosphate groups [7]. Molecular docking of Mg^{2+}-ATP to the ATP-binding cavity in each cryo-EM structure was performed in Schrödinger [8]. For each system, the top-ranked docking pose based on score and visual inspection of interactions was selected for subsequent MD simulations. The complexes were embedded in an explicit TIP3P water model using the *Desmond* MD system builder, neutralized with counterions, and simulated for 100 ns. Trajectories were analyzed for stability, residue interactions, and conformational dynamics of the ATP-binding interface.

Statistical Analysis

Data are presented as mean ± standard deviation (SD) from three independent experiments (N=3). Statistical differences between two groups were evaluated using two-tailed Student's t-test, normality assumptions were verified using the Shapiro-Wilk's test. For comparison between control and experimental groups (more than two groups) was assessed by one-way analysis of variance (ANOVA) followed by post-hoc Dunnett's. A p-value < 0.05 was considered statistically significant. All analyses were conducted using GraphPad Prism version 10.4. Statistics for the cryo-EM data is reported in **Table S1**.

Table S1. Statistics for the determined three cryo-EM structures.

	WT αSyn + ATP	WT αSyn	K21A αSyn + ATP
**Data Collection**			
Pixel size [Å]	0.65	0.65	0.65
Defocus range [µm]	0.8 - 2.4	0.8 - 2.4	0.8 - 2.4
Voltage [kV]	300	300	300
Number of Frames	40	40	40
Total dose [e/Å^2]	63.52	63.12	52.4
**Reconstruction**			
Reconstruction Box width [pixel]	256	256	256
Inter-box distance	33 Å	33 Å	33 Å
Reconstruction Pixel size [Å]	1.3	1.3	1.3
Micrographs	2198	2240	1659
Initially selected segments	552109	1048603	39725
Segments after 2D classification	189918	468631	87149
3D refinement resolution [Å] (FSC>0.143)	3.23	3.53	3.39
Final Resolution [Å] (FSC>0.143)	3.08	3.20	3.13
Estimated map sharpening B-factor [Å]	-104.512	-116.774	-73.778
Axial symmetry	C1		C1
Helical rise [Å]	4.8		2.4
Helical twist [Å]	-0.81		179.63
**Model composition and validation**			
Non-hydrogen atoms (5 layers)	5760	-	4165
Protein residues (5 layers)	840	-	610
R.m.s. deviation, bond length [Å]	0.004	-	0.05
R.m.s. deviation, bond angles [°]	0.962	-	0.960
MolProbity score	1.52	-	1.86
Clashscore	1.62	-	6.56
Rotamer outliers [%]	0	-	0
Ramachandran plot favored [%]	5.43	-	4.75
Ramachandran plot allowed [%]	94.57	-	95.25
Ramachandran plot disallowed [%]	0	-	0
PDB Code	9QYL	-	9QYN
EMDB-ID	EMD-53453	-	EMD-53456

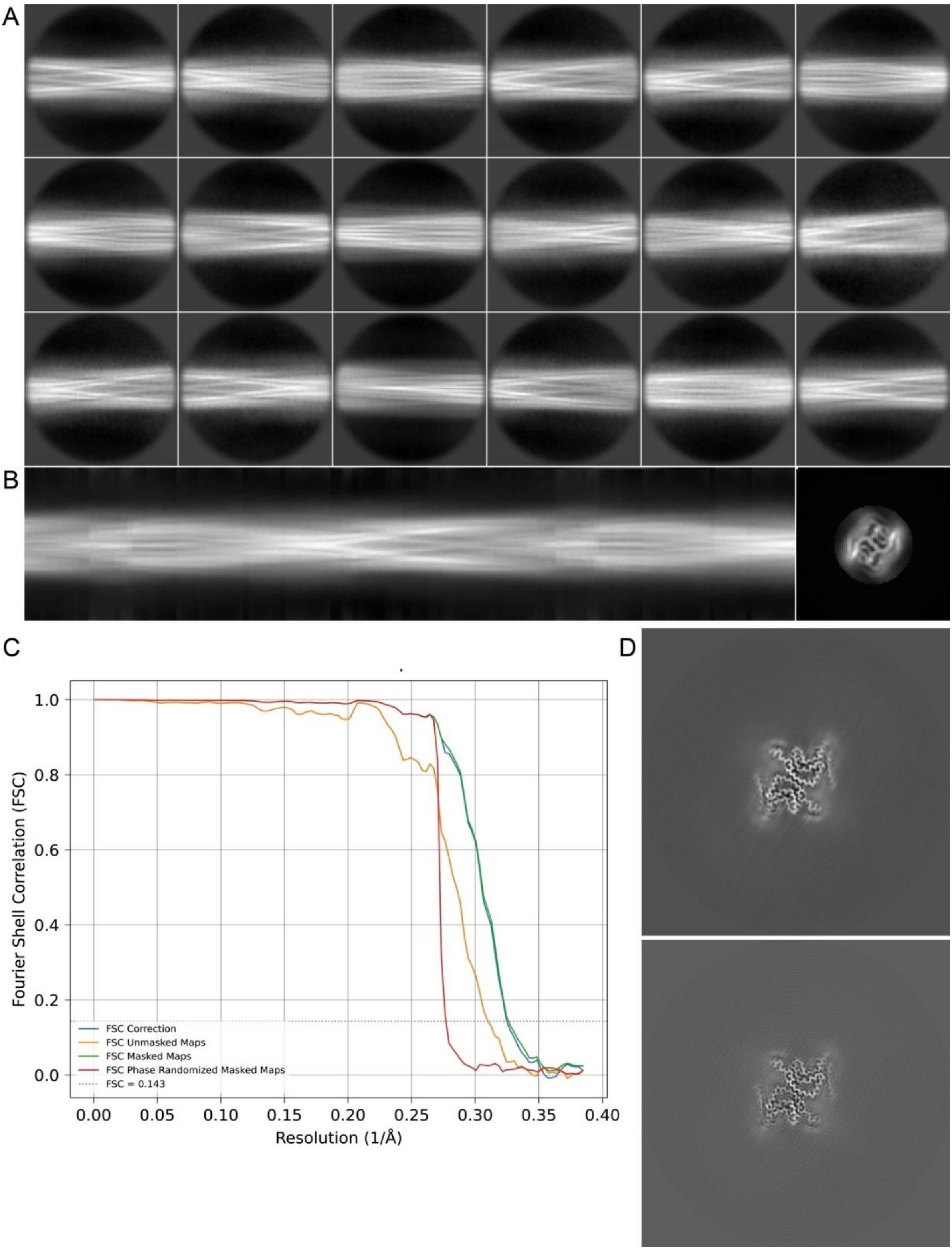

Figure S1. *Processed WT-ATP αSyn cryo-EM data.* A. 2D class averaged images of the fibril segments used to generate the initial model with relion_helix_inimodel2d. B. The output of relion_helix_inimodel2d showing the summed 2D class averages and a z-projection of the reconstructed 3D model used as input for 3D refining. C. The FSC curves generated during post-processing in RELION, where red is the plot for the randomized phase, orange is the unmasked map, green is the masked map and blue is the corrected FSC curve. D. Z-projection of cryo-EM density maps after 3D refinement (top) and postprocessing (bottom).

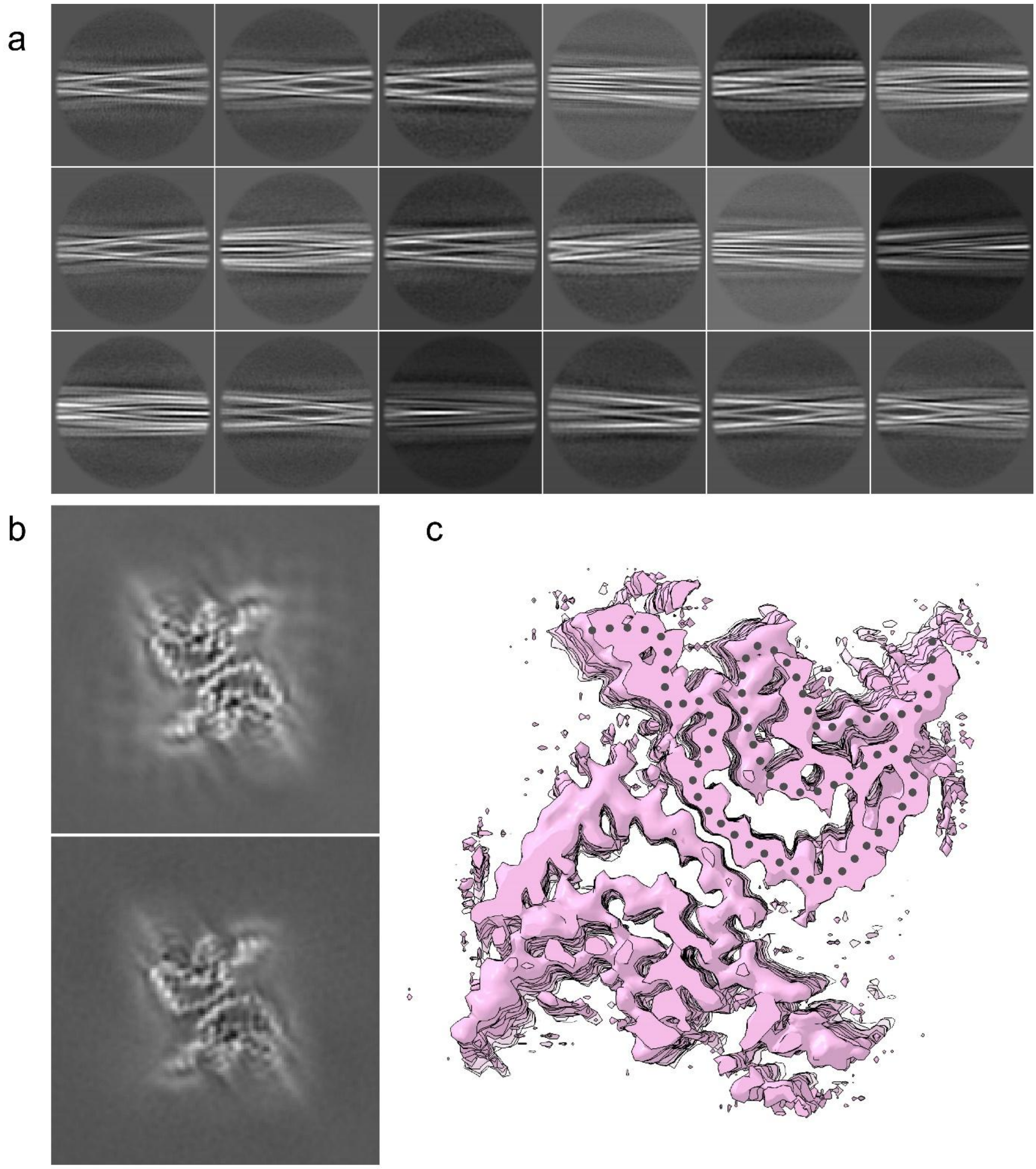

Figure S2. *Helical refinement of WT αSyn amyloids without ATP.* (a) 2D class average images from WT αSyn amyloids without ATP. (b) After multiple rounds of classification and refinement steps, the fibril segments are still not optimally aligned to achieve a high resolution. (c) The 3D refinement resolution map at 3.53 Å (**Table S1**) reveals a mismatch of aligned segments leading to breaks in the Cα trace of the protein's backbone. A hypothetical Cα trace is shown in grey with a dotted line. The amyloid polymorph of the map is clearly similar to type 1A.

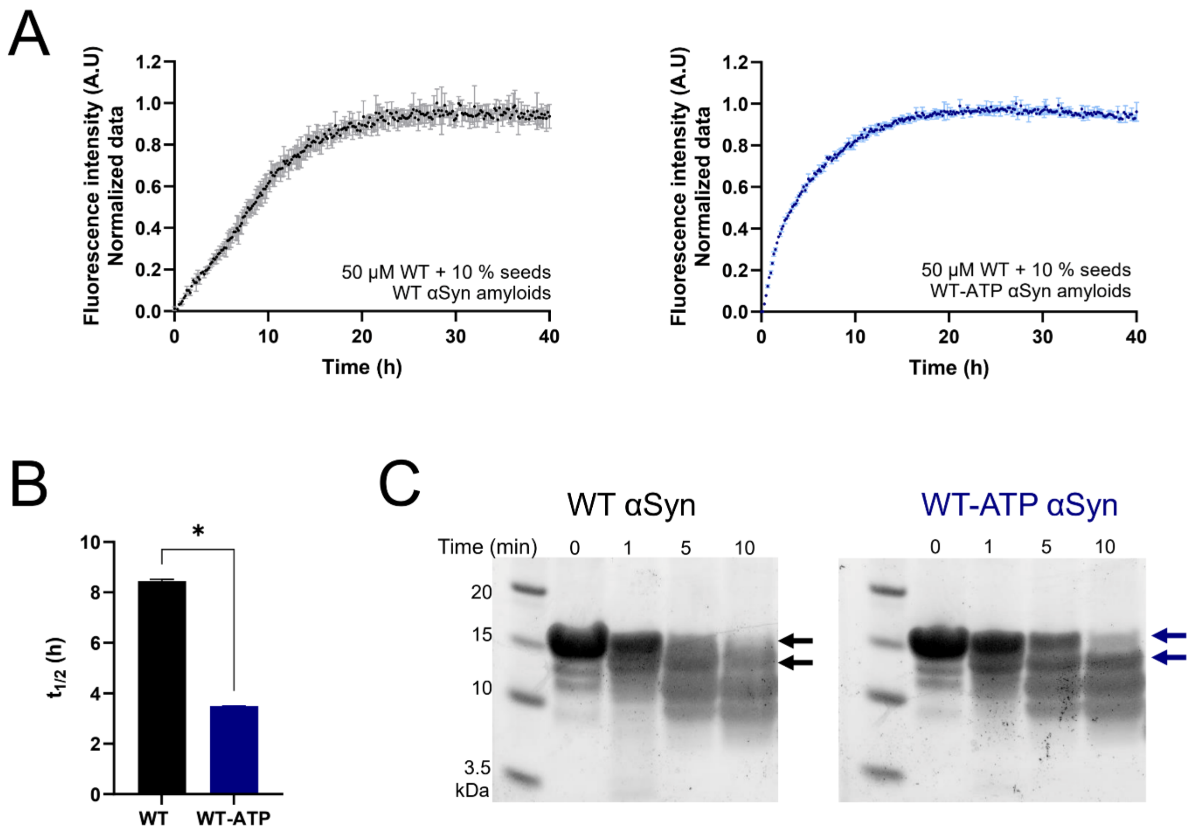

Figure S3: *Seeding ability and proteinase K resistance of WT and WT-ATP αSyn amyloids*. A. Aggregation assay by ThT of 50 µM WT monomers in the presence of 5 µM pre-formed fibrils. B. Aggregation half times ($t_{1/2}$). Error bars, mean ± SD (N = 3). Statistical analysis performed by two-tailed Student's t-test, *$p<0.05$. C. Proteinase K digestion of 50 µM of fibrils at different time points.

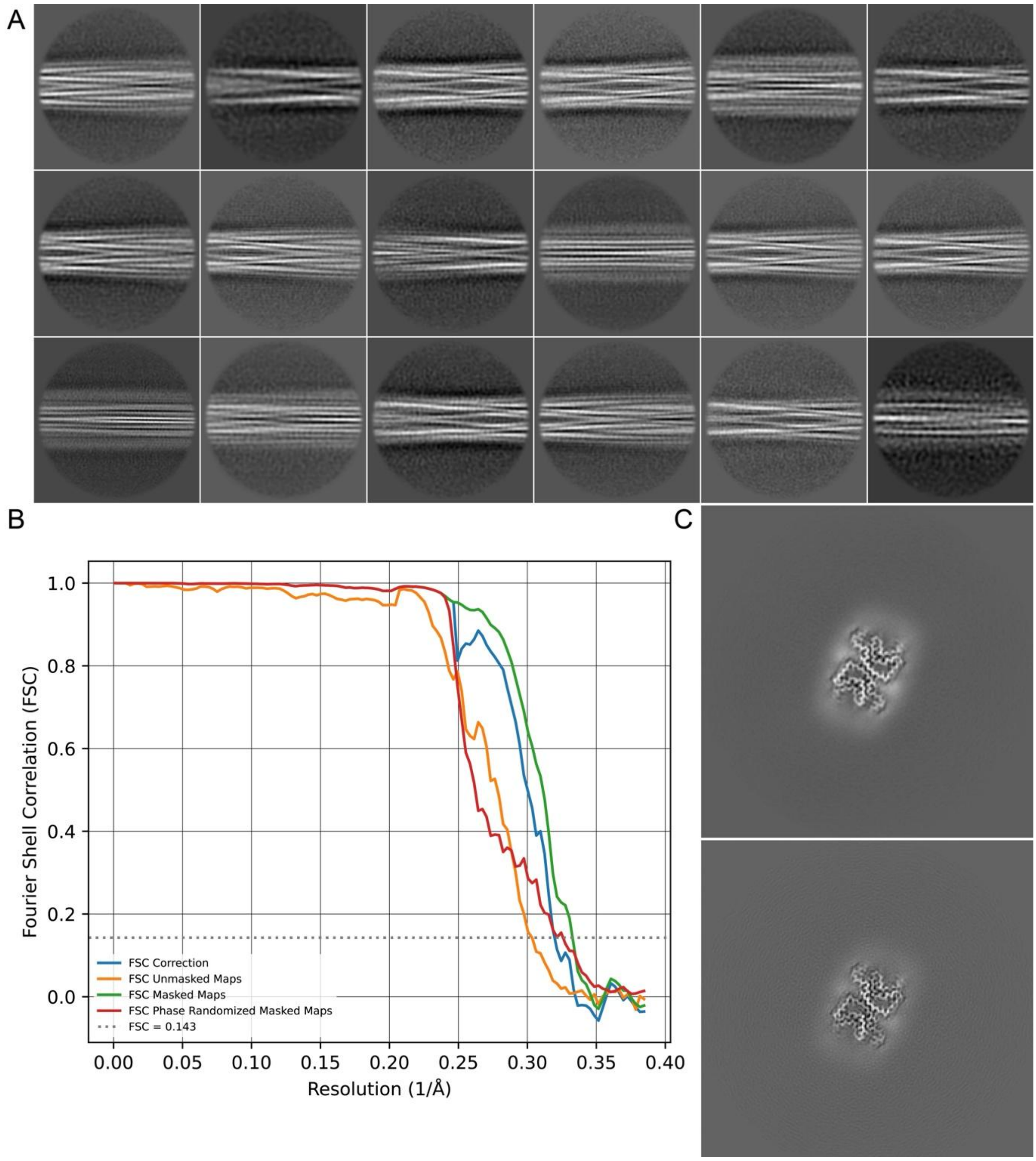

Figure S4. *Processed K21A -ATP αSyn cryo-EM data.* A. 2D class average images of the fibril segments used to generate the initial model with relion_helix_inimodel2d. B. The FSC curves generated during post-processing in RELION, where red is the plot for the randomized phase, orange is the unmasked map, green is the masked map and blue is the corrected FSC curve. C. Z-projection of cryo-EM density maps after 3D refinement (top) and postprocessing (bottom).

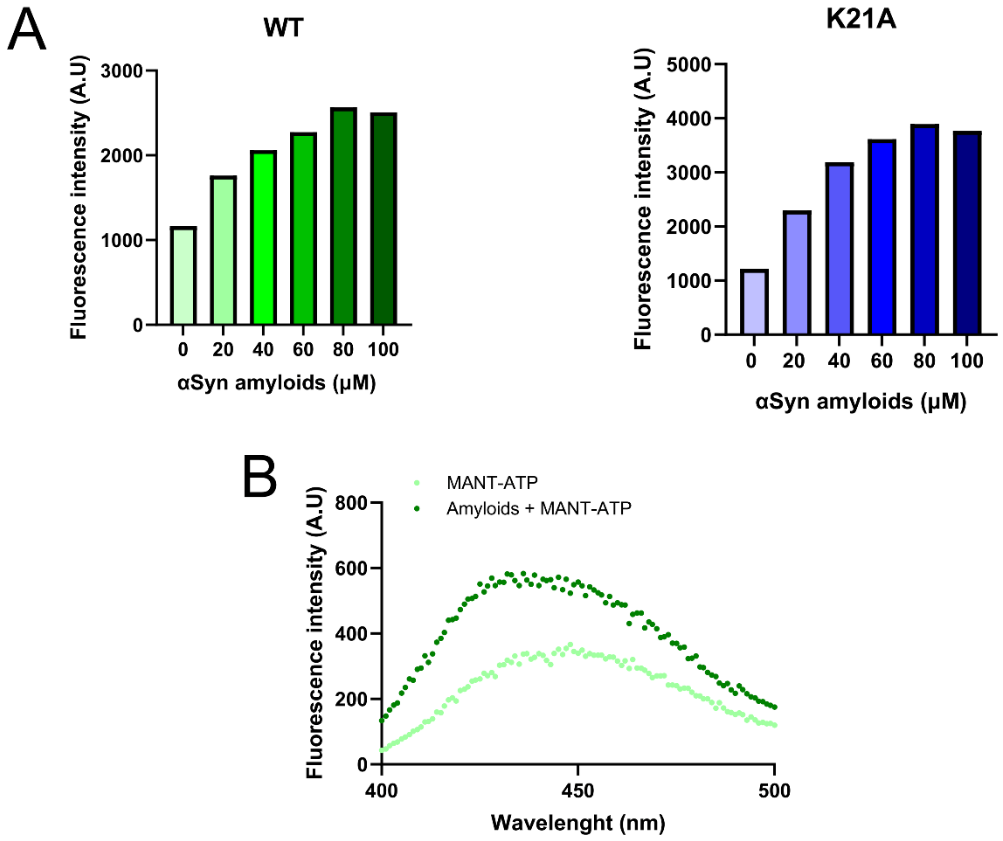

Figure S5. *MANT-ATP binding to αSyn amyloids.* A. Fluorescence intensity of 20 µM MANT-ATP when is binding to different αSyn amyloids concentrations. B. Fluorescence spectra of MANT-ATP in presence or absence of αSyn amyloids.

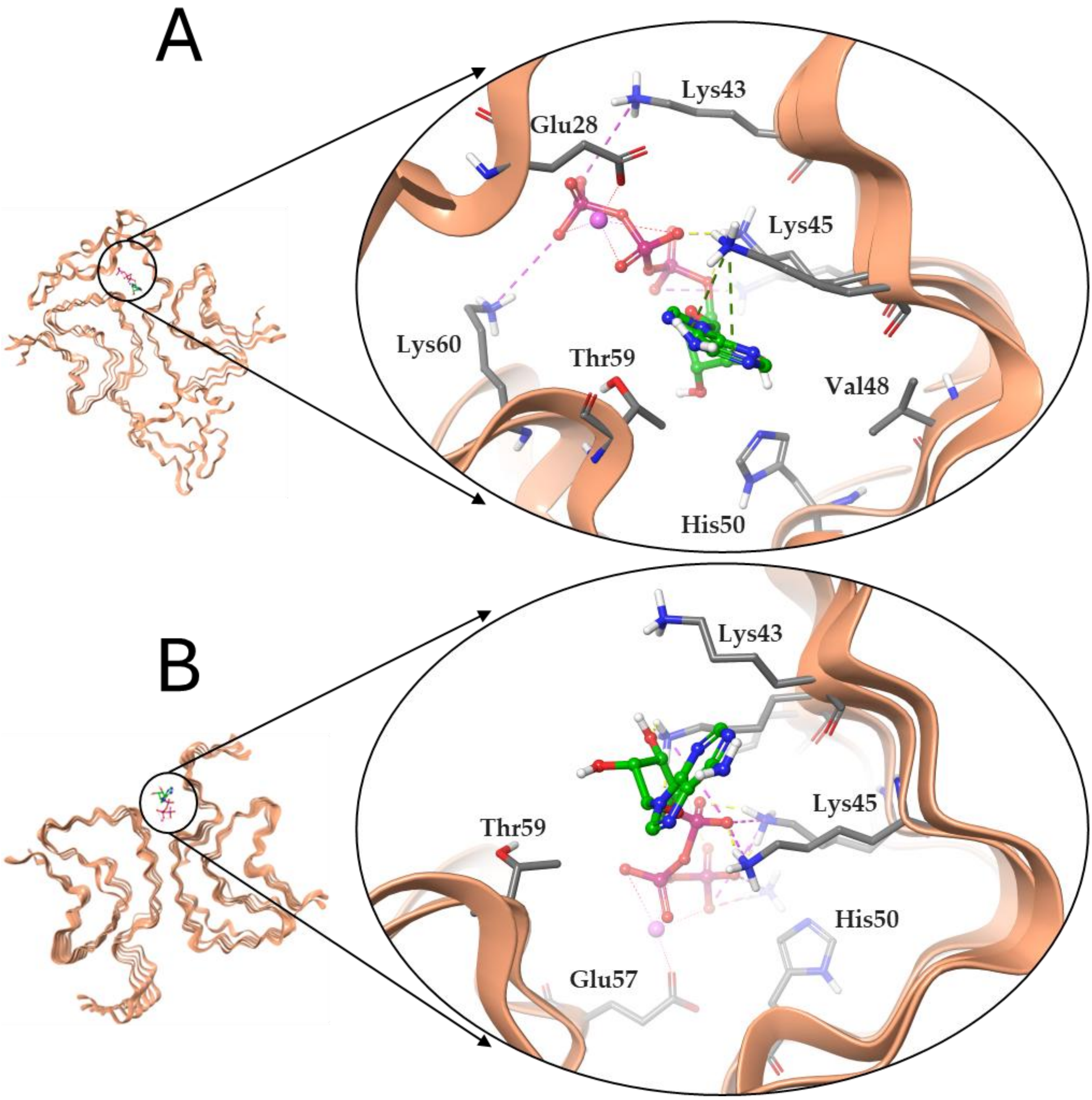

Figure S6. *Docking of ATP to WT and K21A αSyn amyloid structures*. Docked poses of Mg^{2+}-ATP in the cryo-EM models of WT (A) and K21A mutant (B) αSyn. Mg^{2+} is shown as a magenta sphere. Color coding of interactions: hydrogen bonds (yellow dashed lines), cation–π/π–π stacking (pink and green dashed lines). Electrostatic interactions not shown. Mg^{2+} coordination bonds are depicted as fine pink-red dashed lines, representing interactions with the oxygen atoms of the β- and γ-phosphate groups of ATP, and coordinating residues.

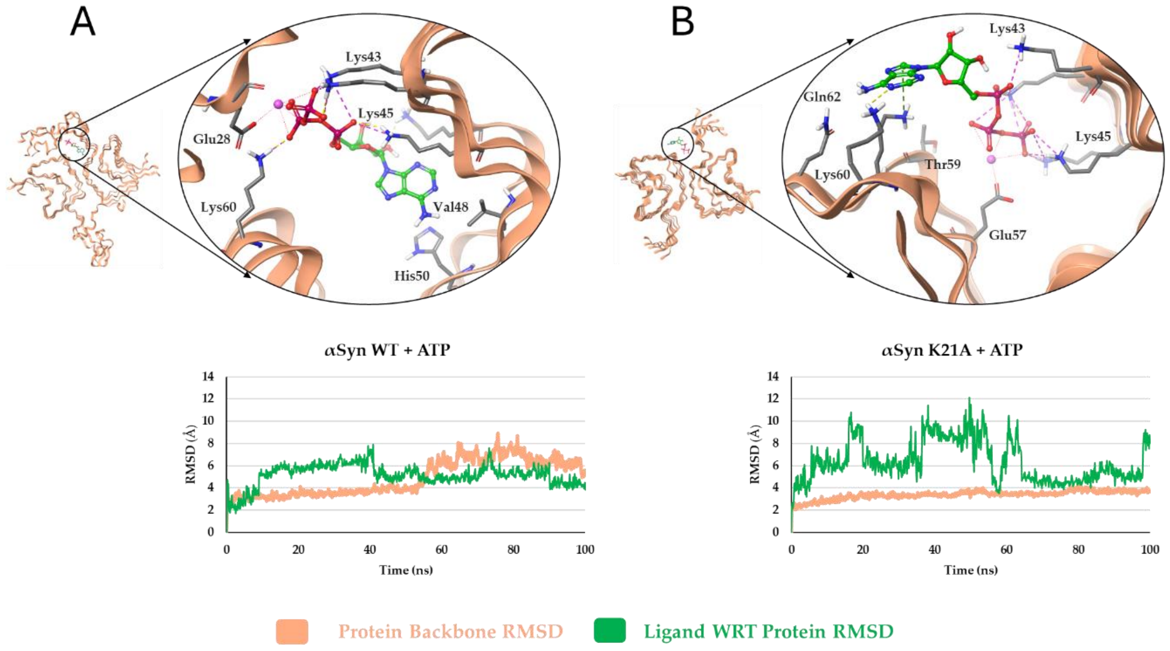

Figure S7. *MD simulations of ATP in complex with WT and K21A αSyn amyloids*. **Top.** Representative ATP-bound conformations from 100 ns MD simulations are shown for WT (A) and K21A (B) αSyn structures with close-up views highlighting the difference in ATP orientation. Mg^{2+}, shown in magenta. Interaction types are indicated as dashed lines: hydrogen bonds (yellow), and cation–π/π–π interactions (pink and green). Electrostatic interactions not shown. Mg^{2+} coordination bonds are depicted as fine pink-red dashed lines, representing interactions with the oxygen atoms of the β- and γ-phosphate groups of ATP, and coordinating residues. **Bottom.** Root mean square deviation (RMSD) plots for protein backbone (orange) and ATP (green) with respect to the protein over the simulation time. Despite local flexibility in the binding region, ATP remains stably associated maintaining distinct orientations throughout both simulations.

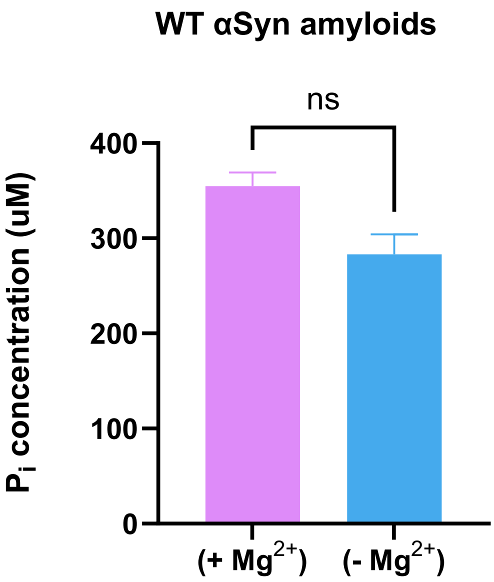

Figure S8. *ATPase activity endpoint at 3 hours*. Inorganic phosphate generated of WT αSyn amyloids incubated with ATP in presence or absence of Mg^{2+} in the reaction buffer. Statistical analysis performed by two-tailed Student's t-test, ns (not significant).

References

[1] A. Burt, B. Toader, R. Warshamanage, A. von Kügelgen, E. Pyle, J. Zivanov, D. Kimanius, T. A. M. Bharat, S. H. W. Scheres, *An image processing pipeline for electron cryo-tomography in RELION-5* **FEBS Open Bio 2024**, *14* (11), 1788, https://doi.org/https://doi.org/10.1002/2211-5463.13873.

[2] J. Elferich, L. Kong, X. Zottig, N. Grigorieff, CTFFIND5 provides improved insight into quality, tilt and thickness of TEM samples. eLife Sciences Publications, Ltd: **2024**.

[3] T. Bepler, K. Kelley, A. J. Noble, B. Berger, *Topaz-Denoise: general deep denoising models for cryoEM and cryoET* **Nature Communications 2020**, *11* (1), 5208, https://doi.org/10.1038/s41467-020-18952-1.

[4] S. Lövestam, S. H. W. Scheres, *High-throughput cryo-EM structure determination of amyloids* **Faraday Discussions 2022**, *240* (0), 243, https://doi.org/10.1039/D2FD00034B.

[5] K. J. Bowers, D. E. Chow, H. Xu, R. O. Dror, M. P. Eastwood, B. A. Gregersen, J. L. Klepeis, I. Kolossvary, M. A. Moraes, F. D. Sacerdoti, J. K. Salmon, Y. Shan, D. E. Shaw, in *SC '06: Proceedings of the 2006 ACM/IEEE Conference on Supercomputing* **2006**, 43-43.

[6] R. C. Johnston, K. Yao, Z. Kaplan, M. Chelliah, K. Leswing, S. Seekins, S. Watts, D. Calkins, J. Chief Elk, S. V. Jerome, M. P. Repasky, J. C. Shelley, *Epik: pK(a) and Protonation State Prediction through Machine Learning* **J Chem Theory Comput 2023**, *19* (8), 2380, https://doi.org/10.1021/acs.jctc.3c00044.

[7] N. G. Holm, *The significance of Mg in prebiotic geochemistry* **Geobiology 2012**, *10* (4), 269, https://doi.org/10.1111/j.1472-4669.2012.00323.x.

[8] S. Parate, F. Buratti, L. A. Eriksson, P. Wittung-Stafshede, *In silico identification of substrate-binding sites in type-1A alpha-synuclein amyloids* **Biophys J 2025**, https://doi.org/10.1016/j.bpj.2025.06.017.
